# Aligning Climate Mitigation, Air Pollution Control, and Healthy Aging

**DOI:** 10.34133/hds.0408

**Published:** 2026-07-03

**Authors:** Huali Wang, Tao Xue

**Affiliations:** ^1^ Dementia Care and Research Center, Peking University Institute of Mental Health (Sixth Hospital), Beijing 100191, China.; ^2^ NHC Key Laboratory for Mental Disorders, National Clinical Research Center for Mental Disorders, Beijing 100191, China.; ^3^ National Institute of Health Data Science and Institute of Medical Technology, Peking University, Beijing 100191, China.; ^4^ Institute of Reproductive and Child Health, National Health Commission Key Laboratory of Reproductive Health and Department of Epidemiology and Biostatistics, and Ministry of Education Key Laboratory of Epidemiology of Major Diseases, School of Public Health, Peking University Health Science Centre, Beijing 100191, China.

Global environmental changes, particularly climate change, persistent air pollution, and extreme weather, are profoundly undermining the well-being of older adults worldwide. This population faces a dual burden: heightened physiological vulnerability and often-diminished adaptive capacity. For instance, chronic exposure to air pollution (e.g., fine particulate matter, PM_2.5_) directly exacerbates cardiovascular and respiratory diseases prevalent in older cohorts [1]. Concurrently, environmental-change-related natural disasters, such as unprecedented storms and floods, frequently disrupt access to critical healthcare and social services, isolating older adults [2]. The impacts extend beyond physical ailments; environmental degradation is increasingly recognized as a driver of broader psychological distress and cognitive impairment [3,4], especially in low-resource communities. This crisis underscores the One Health concept [5], which establishes a critical framework recognizing that human health is inextricably linked to the health of our ecosystems. Improving the environment for older adults is therefore vital for achieving global sustainable development goals. Creating a clean, accessible, and age-friendly environment is essential to ensure their well-being, support healthy aging, reduce health disparities, and promote social inclusion [6].

The evidence base substantiating this link between environmental exposure and the health of older adults is extensive. The physical health of this population is significantly influenced by multiple environmental factors. Long-term exposure to ambient air pollution, for instance, is linked to increased risks of cardiorespiratory diseases [1,7]. Beyond broad epidemiological associations, specific mechanistic pathways are increasingly understood; for example, PM_2.5_ and temperature variability mediate mental and physical health deterioration through the disruption of sleep architecture, the induction of tissue hypoxia, and the triggering of chronic systemic oxidative stress and neuroinflammation. Extreme temperatures, including both heatwaves and cold spells, are also associated with significant rises in morbidity and mortality [8], a challenge exacerbated by population aging [9]. Physiologically, extreme temperature gradients can alter pain thresholds by sensitizing nociceptive C-fibers and transient receptor potential ion channels in older adults with pre-existing conditions, such as disc herniation. These risks are further compounded by the indoor environment, where poor air quality and inadequate heating can complicate chronic disease management. In addition to physical health, these determinants interact with intrinsic age-related vulnerabilities to exacerbate neurodegenerative risks, significantly heightening susceptibility to depression and accelerated cognitive decline [10], which are intrinsically linked to broader metrics of healthy aging, such as maintaining independence [11]. Indeed, a critical demographic paradox exists: The rapid aging of a population, combined with increasing climate volatility, can mathematically override the mortality benefits gained from stringent air pollution reductions. This underscores the extreme urgency of this paradigm shift; emission controls alone are woefully insufficient without concurrent, aggressive improvements in climate resilience and geriatric care. Critically, the evidence moves beyond association; employing quasi-experimental difference-in-differences designs, recent evaluations of China’s Clean Air actions demonstrate that environmental improvements yield tangible, quantifiable co-benefits. For example, specific reductions in PM_2.5_ concentrations have been directly translated into an identifiable delay in the onset of cognitive decline among older adults and significant reductions in household medical expenditures associated with cardiovascular and respiratory hospitalizations [3,12,13].

Despite this clear evidence of co-benefits, achieving synergy requires moving beyond epidemiological association to address the specific bureaucratic, legal, and financial barriers that currently fragment environmental policy from whole-person aged care. At the governance level, intersectoral collaboration remains limited. Overcoming this requires institutionalizing frameworks such as Health-in-All-Policies or the One-Health approach to mandate health impact assessments within urban planning and environmental regulation. Furthermore, executing these integrated policies necessitates innovative funding mechanisms, such as combining environmental protection budgets with aged-care subsidies, or leveraging “green finance” to catalyze age-friendly, low-carbon housing modifications and energy-efficient retrofits. These gaps currently manifest at the societal level, where unplanned urbanization has created heat islands and concentrated air pollution. This macro-challenge filters down to the community and individual levels: At the community level, the living environment is critical, as poor indoor ventilation and a lack of thermal control contribute to respiratory illness; at the individual level, adverse conditions shape risky behaviors, such as promoting sedentary lifestyles when poor air quality limits outdoor activity. Finally, the systemic response to these stressors remains fragmented and uncoordinated. Healthcare delivery is siloed. Older adults with multimorbidity require integrated care, but environmental health is rarely incorporated into geriatric plans. Consequently, the aged-care system itself lacks resilience, remaining ill-prepared for environmental change. This is particularly evident in the gap regarding emergency preparedness and the lack of precise early warning systems for pollution and extreme weather, which are needed to help older adults better manage exercise, activities, and social engagement while protecting their health. This necessitates a shift from siloed interventions to a new paradigm of synergistic planning that can simultaneously address air quality, climate resilience, and healthy aging [14].

**Fig. 1.**
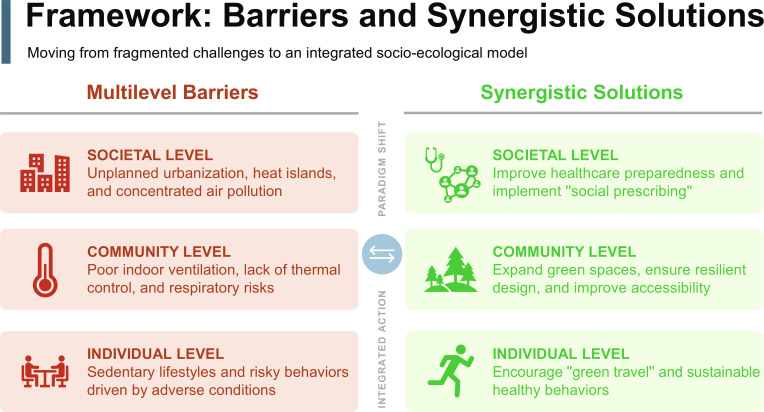
The framework of synergistic solutions to promoting the environment and healthy aging.

Addressing these fragmented barriers and harnessing shared public health mechanisms calls for a fundamental paradigm shift (Fig. 1): from siloed environmental regulation and geriatric care toward an integrated, socio-ecological model of healthy aging that embraces the core principles of “Green Longevity”. Under this framework, older adults are not merely passive victims of environmental change, but are critical, underutilized actors in both mitigation and adaptation efforts. Such synergy can be actualized at the community and individual levels by empowering them to take on roles in community leadership, organize “time-banking” for mutual aid during extreme weather events, participate in local conservation efforts, and facilitate intergenerational education regarding sustainable, low-carbon practices. Furthermore, encouraging healthy and sustainable behaviors, such as “green travel” (e.g., active transport), provides significant co-benefits for both personal health and the planet [15]. Crucially, we must acknowledge that the feasibility of active transport is highly dependent on safe neighborhood infrastructure, which is often systematically lacking in economically deprived or historically segregated areas, necessitating targeted urban planning. At the community level, initiating “green health interventions” necessitates not only expanding accessible green spaces but also optimizing climate-resilient facility design. This ensures that essential services remain accessible during extreme weather, thereby empowering older adults to safely maintain physical exercise and social engagement [16]. Finally, at the societal level, it requires improving the preparedness and accessibility of healthcare services. This includes investing in telehealth infrastructure and digital early warning systems. However, to prevent these technological solutions from widening existing health disparities, mandatory strategies must be implemented to bridge the digital divide. This requires public investments in rural broadband infrastructure, the design of highly intuitive, age-friendly user interfaces, and the funding of community-based digital literacy programs supported by caregivers. Implementing “social prescribing” to connect older adults with community support further bridges healthcare and social needs in response to environmental challenges [17]. Implementing these synergistic interventions is not merely a public health opportunity; it is an ecological and geriatric imperative for the 21st century.
